# Editorial and Miscellaneous

**Published:** 1864-07

**Authors:** 


### EDITORIAL AND MISCELLANEOUS.

*The American Medical Association.*—The 15th Annual Meeting of this organization convened in the city of New York on the 7th of June, and remained in session three days. The attendance was good. It was a pleasant social gathering, and doubtless to those who were there it was a period of needed relaxation from the toilsome routine of the physician's life —but the record of the daily business of the meeting is devoid of interest, and of the value of the communications made through the different sections, a judgment can only be formed when the transactions are in print. The published transactions of the Association do not seem to be so generally circulated as we should think they would be, for we are told by Dr. Wistar, the treasurer, that though the Association numbers above 3,000 members, only 120 copies of the transactions of the 14th Annual Meeting have been disposed of during the past year.

Under the heading of "Hints to Reform," the *American Medical Times* has an article relating to the Association, that is well calculated to excite reflection, and from which we quote as much as we have space:

"During the late session of the National Medical Convention the inquiry was not unfrequently made by the older members—" How can we give more character to the Association and render its meetings more interesting and profitable?" This is, indeed, a most important question, and one that every member who has the welfare of the Association at heart should seriously consider. We have stated that the late meeting was a decided success, and such we must regard it when considered as a pleasant and national reunion of the profession. But when viewed from a higher stand-point, and with a more critical regard to those elements which are to render this body the controlling power in the profession, elevating its moral, social, and educational status, the Association failed to answer the just expectation of its friends. The precious hours of its general sessions were too much occupied with loose discussions of unimportant subjects; while windy, pretentious orators who always float to the surface on such occasions, interrupted the progress of business by points of order, motions, and trivial questions. The sections failed of that degree of interest which they should elicit, owing to the absence of well-prepared papers and searching discussions by members eminent in the department of practice to which such papers belong. But these defects, and others that might be noticed, are not inherent in the Association, and may be corrected if the leading members will take a decided stand in favor of reform. We propose thus early to present this question to the profession, by offering some suggestions, in order to elicit a full and free discussion while the impressions made by the late meeting are still fresh in the minds of members.

The Association has properly a two-fold character; it is both an ethical and a scientific body, and it is very important to maintain these features in full strength and harmony. But there is danger of its losing both, and gradually sinking into hopeless imbecility. It is essential to the integrity and good government of the profession that there be some great central organization to which it may refer all questions disturbing its relations or affecting its general welfare. It is not less important that we have a great central scientific body which shall be the patron of the medical sciences, and shall stand before the world as the representative of our national medical literature. To both of these positions the Association may attain if discreetly managed.

"To become the ethical or governing body of the profession one thing above all others is requisite, viz., that the Association command the respect of the profession. It can not be the arbiter of medical opinion, nor the instructor in morals and professional amenit es, without itself being above reproach. And as a corrupt body can never be purer and better than the individual members, the Association must, if it would place itself upon the highest ground, purge itself of those blighting excrescences which cling to it as to their last and only hope of respectability. In other words, the Association must have a more select representation. At present medical societies exercise too little discretion in the selection of delegates; the position is given to any one who volunteers to attend, and such volunteer is generally the "wind bag" of the Society. The Association should limit its representation to State, County, and well established, legally incorporated bodies, and exclude those ephemeral voluntary organizations which too often exist only to enable some objectionable person to gain a membership. It would do well to establish a title of membership; and, placing a high qualification upon it, subject its list of permanent members to a searching revision. Names stand recorded on that roll which are no longer worthy of such associations. If by such or any other means the representatives of the profession could be more select, embracing only the better cl ss, the meetings of the Association would be dignified, its discussions deliberate, and its decisions would command respect."

The officers for the ensuing year are

*President*—N. S. Davis, of Illinois.

*Vice Presidents*—W. S. Mussey, of Ohio; Worthington Hooker, of Conn.; Wm. Wheelin, of Ind.; F. E. B. Heintze, of Maryland.

*Permanent Secretary*—Wm. B. Atkinson, of Penn.

*Assistaut Secretary*—H. R. Storer, of Mass.

*Treasurer*—Casper Wistar, of Penn.

Prize Essay by S. Fleet Speir, on the Pathology of Jaundice. The next annual meeting will be held in Boston.

---

*The Growth of Bone.*—M. DeLamballe, in his researches on the generation and reproduction of tissues, corroborates the assertions of M. Flourens. 1st. That bones increase in thickness by external and superimposed layers. 2d. That they increase in length by the addition of terminal layers arranged in juxtaposition. 3d. That proportionately as the new layers are deposited externally, the older ones on the inner surface are re-absorbed. 4th. That ossification consists in the regular and successive conversion of periosteum into cartilage, and cartilage into bone.

---

*Hydrophobia in Dogs.*—M. Leblac has revived the old theory that "restraint from sexual intercourse" is a potent cause of hydrophobia in the dog. In support of which, he says, "On one side of a portion of the Danube the Christians have none but male dogs, whereas on the other side the Turks leave the animals of both sexes in a state of absolute freedom. On the Christian side hydrophobia is frequent. On the other, unknown." It ought to be unknown in this country.

---

*Tetanus in the Army.*—Tetanus is prevailing among the wounded of the Army of the Potomac to an unusual extent, nearly every case proving rapidly fatal. Dr. E. Brown-Sequard, we are glad to learn, has consented to give a few lectures on this disease, at Washington, where it is most prevalent.

---

Most of the Medical Journals have, or intend advancing their price of subscription.

---

*Two Deaths from Laughing Gas.*—A short time since a merchant of Philadelphia, Mr. Sears, died from the effects of laughing gas, administered for the painless extraction of a tooth. A traveling dentist, at a public exhibition of laughing gas, at Swanton Falls, Vt., administered this gas to several persons. Among the number was a beautiful girl, seventeen years of age, the daughter of W. H. Bell, a highly respectable citizen. The day after inhaling the gas she was taken ill, and died the following day from its effects.

---

*Arsenic in Pemphigus.*—Mr. Hutchinson, of the Metropolitan Free Hospital, speaks very highly of the use of arsenic in pemphigus. He says that "it renders relapses less likely, and that an improvement may be noticed immediately on its employment, not a single fresh bulla showing itself after the first few days of treatment.

---

*Prolongation of Anæsthesia by Chloroform.*—Drs. Erlenmeyer and Nussbaum state that by subcutaneous injection of one-eighth grain doses of sulph. morphiæ the anæsthesia produced by chloroform may be protracted without danger, the patient passing at once into a sound sleep of several hours' duration. It is an experiment well worthy of trial in protracted operations, and can be easily performed with one of Ames' needle pointed syringes.

---

*Garibaldi in England.*—One of the principal reasons for Garibaldi visiting England was to place himself under the care of Mr. Ferguson, of London. His general condition is declared to be excellent.

---

*Sulphur in Asthma.*—M. Ducles, of Tours, recommends this substance in doses of three fourths to one and a half grs. three times a day for several months. The Boston *Med. and Surg. Journal* mentions three severe cases where asthma was completely cured by this very simple treatment.

---

*Agreeable Mode of Taking Senna.*—Dr. Lithner (Buckner's Reportorium,) says that senna leaves (one or two drachms to one or two cups of water,) should be allowed to infuse all night in cold water. With the strained infusion coffee is prepared next morning as if with water, and an aperient which does not taste of senna, and does not cause griping, is thus produced.

---

*Collodium for the Stings of Wasps.*—Dr. Munde, in the *Lancet*, states that a coating of collodium applied to the part injured causes the pain to disappear and the swelling to rapidly subside.

---

*Pennsylvania Hospital.*—Dr. James Pancoast has resigned his situation as one of the surgeons of this institution, and Dr. Thomas George Martin has been selected in his place.

---

*Saracenia Purpu[illegible]ea.*—Dr. James Watson has experimented in eight cases of small pox, in the Royal Infirmary, with this newly vaunted Canadian remedy for small pox, and found it absolutely inert.

*Honey as an Excipient for Pills.*—M. Thibault (*Bulletin de Thérapeutique,*) believes that much of the disappointment following the employment of pills arises from their, as ordinarily prepared, acquiring a degree of induration that prevents their solution, and enables them to traverse the alimentary canal unchanged. To prevent this he recommends the employment of honey, as pills prepared with it always remain soft, however long they may be kept.

---

The Iodide of Lime as a substitute for Iodide of Potassium is rapidly gaining favor among distinguished London physicians. It is used in those cases where Iodide of Potassium is indicated, with more marked effects than usually attend the use of that salt. The lime and iodine are held together by a very feeble affinity, and the salt will not admit of exposure without evolving free iodine. ·The solution is a colorless and almost tasteless liquid, and remains permanent, although long kept and exposed to the air.

Each drachm of the salt contains eight and a half grains of iodine. The Iodide of Lime is superior to Iodide of Potassium in several particulars, as, 1st. The smallness of the dose, and the minute state of its atomic division. 2d. In not passing off so quickly through the kidneys. 3d. In its ready combination with the blood and tissues, manifested by its alterative effects. 4th. In being nearly tasteless, and therefore more readily taken, especially by children. 5th. In being twenty times less expensive. 6th. In not producing either gastro-enteric or vesical irritation. The dose of the salt is about one-fourth of a grain, given in solution three times a day.

